# Delivering heat-resilient housing in England: Reflections on the role of overheating building regulations in a warming climate

**DOI:** 10.1016/j.enpol.2026.115152

**Published:** 2026-02-17

**Authors:** S. Halai, N. Hughes, C.H. Simpson, A. Mavrogianni, M. Davies

**Affiliations:** a University College London, Institute for Environmental Design and Engineering, Central House, 14 Upper Woburn Place, London, WC1H 0NN, United Kingdom; b University College London, Institute for Sustainable Resources, Central House, 14 Upper Woburn Place, London, WC1H 0NN, United Kingdom

**Keywords:** Building regulations, Part O, Cooling, Overheating, Climate adaptation, Heat resilience

## Abstract

Climate change is causing a rise in global temperatures. High summertime temperatures can lead to overheating, where the temperature in homes becomes a risk to health and comfort. To reduce overheating in new residential buildings, a new part of the English Building Regulations, Part O: Overheating (‘Part O’) was introduced in 2022.

This study evaluates the application of this new regulation to real-world projects. Semi-structured interviews were carried out with 30 experts across the construction and policy sectors. Experts identified that Part O has barriers to its application, including conflicts with broader parts of the building regulations, limitations in compliance modelling assumptions for passive and active technologies, a lack of futureproofing for rising temperatures, and inadequate assurance that homes designed to the regulations would be constructed to a high quality.

To improve the use of Part O, this paper suggests recommendations for how the building regulations can minimise conflict, streamline processes, and support innovation to deliver heat resilient homes. This includes using academic evidence to better support technical modelling assumptions, and in the longer-term utilising upcoming modelling methodologies (Home Energy Model) to integrate Part O modelling with broader parts of building design and compliance requirements. To deliver a shift in high quality, heat resilient and net zero homes, this paper recommends a long-term shift in regulatory approaches to focus on absolute performance outputs, building audits and post occupancy evaluation.

## 1 Introduction

Anthropogenic emissions are causing global surface temperatures to increase (IPCC, 2023). Perkins-Kirkpatrick and Lewis (2020) show heat events are increasing in frequency, intensity and duration, globally since the 1950s. In England, the frequency of hot spells is increasing, with projections showing summertime temperature may increase by 6.8 °C in a high emissions scenario (Met Office, 2022).

Rising temperatures can cause overheating. This is when temperatures within buildings increase beyond a comfortable limit impacting on the health and wellbeing of occupants (CIBSE, 2017). Excess heat exposure, when unmanaged, can lead to heat stroke, reduced sleep quality, reduced concentration and productivity, and in extreme cases, mortality (WHO, 2024). Across Europe, the 2022 heatwave is associated with approximately 60,000 mortalities (Beck et al., 2024). It was estimated the same heatwave episode led to 3000 excess deaths in England (UKHSA, 2025). As summertime temperatures continue to rise, without adaptation, this number could exceed 10,000 per year by 2050 in a high emissions scenario (Murage et al., 2024).

Adapting the UK housing stock for higher summer temperatures can manage indoor temperatures to provide safer, and more comfortable indoor environments. Methods to protect from overheating within homes include passive design interventions, active technologies and behavioural or lifestyle changes. Passive technologies include installing shading such as shutters, blinds or eaves, and introducing natural ventilation during cooler periods of the day. Active technologies include mechanical systems such as fans, forced ventilation, or air conditioning. Lifestyle changes might include adapting daily routines, minimising heat producing appliances in homes, adjusting clothing, reducing activity levels or finding cooler spaces to spend time during warmer periods of the day.

In recent years, local planning policy has required housebuilders to show how new developments address overheating risk. The Greater London Authority, for example, require planning applications to show how buildings address the ‘cooling hierarchy’, ensuring applicants use passive measures to minimise overheating risk first, before proposing active cooling (Greater London Authority, 2021). This has been included within other planning requirements outside London, such as Cambridge City Council (Cambridge City Council, 2021), as a method to ensure new homes are protected from rising temperatures.

More recently, a new part of the building regulations for England, Part O: Overheating, was published along with an Approved Document: Part O (‘Part O’) (2021). The regulations were published in 2021 and came into force in June 2022. Part O sets standards to limit overheating in new build homes. The regulations support passive technologies to “limit unwanted solar gains in the summer” and therefore manage overheating. However, they do not rule out the potential for cooling, stating “mechanical cooling may only be used where insufficient heat is capable of being removed from the indoor environment without it” (Part O, 2021). Part O forms just one regulatory document within a complex system of building regulations. The regulations cover building structure, fire safety, water use, ventilation, noise, fuel and power, accessibility, electrical safety and security, amongst other building design requirements.

This new part to the building regulations is a promising step towards adapting how homes are built and to protect residents against overheating. Like with all policy interventions, evaluating the performance of the new regulations is an important part of improving later versions (HM Treasury, 2020). In 2023, the Department of Levelling Up, Housing & Communities (DLUHC), now the Ministry of Housing, Communities and Local Government (MHCLG), called for evidence on the application of Part O (FHS, 2023). This study therefore adds to this evidence base to support future policy changes which can improve the application and use of Part O to deliver overheating-resilient homes.

To evaluate the use of Part O, this study presents semi-structured interviews with 30 experts in the construction and policy sectors. These experts include designers, contractors, housing associations, housebuilders, housing insurance providers, industry bodies and policymakers at local and national government. The aim is to understand socio-technical barriers experts in the construction industry face in delivering overheating-resilient homes on real-world projects. As a result, this paper recommends some suggestions for improving the building regulations to address the barriers faced within the industry.

This paper first sets out a contextual overview of the policy documents relevant to overheating. It also summarises upcoming building regulations and policy for England, which may impact on the overheating requirements. The interview results are then presented. They include a summary of issues participants found in applying Part O to their projects, as well as the broader application of Part O among other parts of the building regulations and the building delivery process. The discussion includes observations from the interview findings, and recommendations on how the application of Part O can be improved to ensure homes are well adapted for a warming climate.

## 2 Context

The Building Regulations: Schedule 1 (2010), for England, sets out minimum design requirements in multiple ‘Parts’ of the regulations. Some Parts to the building regulations ensure immediate safety (e.g. Part A: Structure, Part B: Fire Safety), while others look to inform long-term health such as Part O: Overheating, or sustainability such as Part L: Conservation of Fuel and Power. Each ‘Part’ of the regulations is published with accompanying guidance, referred to as the ‘Approved Documents’, which “give practical guidance … on how to meet the requirements for the Building Regulations 2010 for England” (Part O, 2021). Approved documents recommend technical design standards and modelling methodologies which will comply with the building regulations, but state that “there may be other ways to comply with the requirements than those described in an approved document” (Part O, 2021).

The regulations are enforced by planners as well as building control to uphold a minimum level of quality in building delivery. The role of planners and building control were introduced in the Housing, Town Planning Act in 1909 which established planning and building control as two separate functions (UK Parliament, 1909). Today, planners, sit within local authorities; they approve developments according to their local needs, and ensure the design is compliant with the regulations. The role of building control is to check that completed buildings meet the requirements set by the building regulations. Historically building control was a responsibility of local authorities, however this role was partially privatised in the 1984 Building Act (Ley, 2000).

Part O was introduced in 2021, and came into force from June 2022 (Part O, 2021). It requires that “reasonable provision must be made in respect of a dwelling, institution or any other building containing one or more rooms for residential purposes, to (1a) limit unwanted solar gains in summer; (1b) provide an adequate means to remove heat from the indoor environment.” In meeting this requirement, it further states that “(2a) account must be taken of the safety of any occupant, and their reasonable enjoyment of the residence; and (2b) mechanical cooling may only be used where insufficient heat is capable of being removed from the indoor environment without it” (Part O, 2021).

Part O was published with the Approved Document: Part O (‘Part O’). It includes two approaches to meet building compliance for new dwellings only, a “simplified method” and a “dynamic thermal modelling method” (Part O, 2021). The simplified approach sets dimensions and limits on certain aspects of the building design including the size of glazing, the maximum opening dimensions of glazing, location of windows and minimum free area of window openings for ventilation, and availability of secure openings. It also includes methods to block solar gains using shading such as external shutters and overhangs, and limiting incoming solar gains by specifying tinted windows. So long as dimensions and designs meet these specifications, evidence of dynamic simulation modelling is not required to comply with the regulation.

Alternatively, the dynamic thermal modelling method “may offer the designer additional design flexibility over the solutions in the simplified method” (Part O, 2021). To demonstrate compliance, modelling against a methodology called Technical Memorandum 59^1^ (TM59) published by the Chartered Institute of Building Services Engineers (CIBSE) should be used. TM59 sets out definitions for modelling assumptions and criteria that quantify adequate thermal comfort (CIBSE, 2017). It is recommended those applying for planning and seeking building control approval use this modelling methodology to show how new dwellings comply with the overheating requirements.

TM59 was a well-known methodology within the industry before its introduction for compliance with Part O. It was published in 2017, following the publication of a similar modelling methodology (Technical Memorandum 52^2^) in 2013, a methodology to assess overheating in non-domestic buildings. They have both subsequently been used to inform local planning policy and design standards such as the Building Research Establishment Environmental Assessment Model (BREEAM, 2025). TM59 describes itself as “a standardised approach to predicting overheating risk in residential building designs (for new build and major refurbishments) using dynamic thermal analysis” (CIBSE, 2017, p. 1). It goes on to explain the “methodology is intended for use by designers to influence building design for the better”. To comply with the TM59 criteria, and therefore Part O, modellers must show that living rooms, kitchens and bedrooms do not exceed an average temperature difference between inside and outside by more than 1 °C for more than 3% of the occupied hours. In addition, bedrooms should not exceed 26 °C for more than 1% of annual hours (CIBSE, 2017, p. 5). For homes with restricted window openings “all occupied rooms should not exceed an operative temperature of 26 °C for more than 3% of annual occupied hours” (CIBSE, 2017, p. 5).

The approach that TM59 takes to assess compliance is by setting modelling assumption profiles for occupancy profiles and internal gains profiles. This captures how occupants use the rooms, and how equipment and activities (internal gains) increase internal temperatures within living spaces over a day. The consistency in profiles tested across all homes provides a way of comparing performance across multiple dwellings to a common standard. For compliance with TM59, dwellings must be modelled against a DSY 2020 high emissions 50th percentile scenario. This is a Design Summer Year (‘DSY’) weather file which is based on the UK climate projections (UKCP09^3^) medium emissions scenario and assumes a moderately warm summer, which is the most probabilistically likely to occur. Weather files for 2020, 2050 and 2080 are all available to represent modelled rising temperatures for alternative summer scenarios (a moderate heat year, an intense heat events and a long heat event), for different locations across the UK, and for different likelihoods of occurrence (CIBSE, 2025a,b). Weather files required for Part O compliance were created in 2016 using UKCP09 climate projections (CIBSE, 2009; Virk and Eames, 2016). The weather files have also recently been revised for 2030, 2050 and 2080 weather data using UKCP18^4^ climate projections (CIBSE, 2025a,b).

Part O interacts with many other parts of the building regulations, listing “interactions” with Part B (Fire Safety), Part F (Ventilation), Part J (Combustion appliances and fuel storage), Part L (Conservation of Fuel and Power), Part K (Falling, Collision and Impact), Part M (Access to and use of buildings) and Part Q (Security in dwellings) (Part O, 2021, pp. 2–3). By interactions, this refers to the possible trade-offs and conflicts in building design as a result of multiple regulatory requirements. For example, window glazing ratios and openings sizes may be limited by Part F for ventilation reasons, Part O for overheating reasons, Part L for energy efficiency and heating reasons, Part K for health and safety reasons and Part Q for security.

Both Part O and Part L recommend that buildings are assessed against a compliance modelling methodology during design stage. Part L currently uses SAP (Standard Assessment Procedure) to calculate the regulated energy demand of a building, this is energy which is attributed to the building services, it includes heating, cooling, hot water, lighting, pumps and fans (Part L, 2021). It does not include plug-in devices, such as cooking and televisions (BEIS, 2013). It is important to note that both SAP and TM59 make standardised assumptions for how people use homes including how they occupy rooms, their comfort levels and typical efficiency assumptions associated with some technologies. As such, they are not reflective of how all residents use their homes, but they are intended to be a method to compare buildings against one another.

Part L also requires homes to show compliance modelling to create an Energy Performance Certificate (EPC) which uses SAP calculations to provide an energy rating (A to G) for homes. In England, EPCs are required when a building is newly constructed, sold or let (MHCLG and DLUHC, 2021). This was introduced following the EU^5^ Energy Perfor- mance of Building Directive which requires energy performance certificates for homes across Europe (EPBD, 2002). The EPC approach has since been used to improve building performance of existing buildings across England (DCLG, 2017).

Part O was released with a new iteration of Part L and Part F as part of a step change towards the Future Homes Standard (‘FHS’) (FHS, 2023). The FHS sets draft design standards for how buildings must be designed and delivered to meet compliance with future building regulations. In general, the FHS shifts towards improved building fabric, towards more efficient heat technologies and away from natural gas for heating. The draft guidance proposes improved U-values and reduced thermal bridging, and the use of energy efficient and low carbon technologies such as mechanical ventilation heat recovery (MVHR) and heat pumps. With the new FHS, and Part L, a new compliance modelling approach called the Home Energy Model (HEM) is set to be released. The aim is for HEM to eventually supersede SAP to provide a new modelling approach for Part L compliance to address some of the challenges in using SAP. These challenges include SAP’s crude standardised modelling assumptions associated with occupants and technologies, its calculation approach, the lack of transparency, and its inability to represent new, innovative technologies (HEM, 2023a). At present, it is proposed that HEM will not be used to assess overheating risk, only to model the regulated energy use in homes. However, the calculation methodology allows for detailed overheating calculations including the input of shading, DSY weather files, the representation of dynamic solar gains and internal gains and detailed occupancy profiles (HEM, 2023b).

## 3 Methodology

To evaluate the application of Part O, interviews were conducted with experts in the construction and policy sectors. The sample of interviewees were chosen to extract a variety of viewpoints. By documenting these views, they can be used as an evidence base to support further research and action in heat resilience.

Semi-structured interviews were used to allow for a “flexible and fluid structure” which “can be shaped by the interviewee’s own understanding as well as the researchers, interests” (Lewis-Beck, Bryman and Liao, 2003). This approach allows for “unexpected themes to emerge” and for interviewees to express their “knowledge, understanding, interpretations, experience and interactions” of the problem (Lewis-Beck, Bryman and Liao, 2003). This method has been used in previous studies to explore social aspects of complex socio-technical problems in energy and buildings (Dadzie et al., 2018; Li and Pye, 2018; Hoggett et al., 2024; Howarth and Robinson, 2025).

A purposive sampling strategy was employed to identify and gather stakeholders based on their connection to overheating, retrofit, energy and buildings and capture a variety of viewpoints and experiences. For qualitative studies, this approach provides an “efficient” yet “information-rich” data source (Ritchie et al., 2024). Due to the rich data of interviews, in comparison to surveys for example, Ritchie et al. (2024) suggests keeping the sample small, to manage the complexity of the analytic task and to allow in-depth analysis of the material gathered. An initial set of seven interviews were carried out based on this sampling approach. A snowballing technique was then used to identify new participants to cover contrasting or new viewpoints. Snowballing is when new interviewees are identified based on references or perceptions about other actors relevant to the research question, as well as recommendations from interviewees. Interviews were finalised when the interview data reached saturation, this is the point at which no new themes were being identified (Lewis-Beck, Bryman and Liao, 2003).

Since the research was focused on the English regulations, the sample of professionals were based in England. Thirty participants were interviewed over a total of twenty-four interviews which were conducted between 2023 and 2024. Participants came from a variety of backgrounds including housebuilders, housing insurance providers, housing associations, contractors, designers, cooling manufacturers, charities, social researchers, local government and national government. A summary of interviewees and their sectors are provided in Table 1. Interviews were all carried out by the primary researcher which were recorded, anonymised and transcribed using Microsoft Teams. The transcription was reviewed and coded using nVivo 14.0 (Lumivero, 2023), which allowed for analysis of coding coverage. An inductive analysis was carried out, in that codes and themes were identified from the interview data without any preconceived notions (Erlingsson and Brysiewicz, 2017).

Thematic saturation was assessed iteratively during the coding process, with no substantively new codes or themes emerging in the later stages of analysis, suggesting adequate coverage of the dataset (Olmos-Vega et al., 2023). All interviews were coded by the primary researcher, ensuring consistency in the application of codes, although increasing the risk of bias. Throughout the analysis, reflexivity was maintained by the primary researcher through critical reflection. The primary researcher’s background in construction may have enabled more technical insights in the discussion and application of Part O, however, this likely led to biases in the emerging themes. To overcome this, the emerging themes were discussed with the wider research team to interrogate themes, refine interpretations and deliver a more robust analysis.

## 4 Results

All participants approached for this research indicated that both existing and new homes were at risk of overheating, both in the present and future. In general, there was a consensus that Part O was welcome and needed to drive forward design against overheating risk *(GUI1, CON1, HBUIL1, HBUIL3, INS1, MANF1)*. One participant said the introduction of Part O in the building regulations *“has raised overheating risk up the agenda” (CON1)*, another stated *“it’s good, because it drives action” (MANF3)*. However, many perceived that the current motivation from government (including policymakers themselves) prioritised climate mitigation over climate adaptation *(NPOL1, LPOL2, NPOL3, LPOL4, GUI1)*. One participant described climate adaptation as *“always playing second fiddle” (LPOL4)*. Another interviewee explained, overheating design is often deprioritised in new build housing delivery, *“of the homes which are being addressed, most of them consider climate mitigation but rarely adaptation” (GUI1)*.

Of the 30 interviewees, 19 had engaged with and used the Part O regulations in their work. They identified some issues in its roll out and application on their projects. This section sets out observations made by experts within both the construction and policy sector in the application of Part O. In doing so, it identifies areas for action and improvement for the next iteration of the Part O regulations. The three key themes identified are:

1. Improved assumptions and modelling approach to support passive technologies
2. More futureproofing for rising temperatures in both new build and existing housing stock
3. Flexibility and streamlining to overcome conflict and deliver high quality homes

### 4.1 Improved assumptions and modelling approach to support passive technologies

Many participants used terms such as *“TM59”* and *“modelling”*, suggesting that they used the dynamic thermal modelling approach, rather than the simplified method, to demonstrate compliance with the Part O regulations. In the application of the dynamic thermal modelling approach, interviewees expressed concerns over how *“unrealistic”* and *“stringent”* the modelling assumptions were. By this, the interviewees were explaining how the modelling assumptions did not represent real-world homes and occupants. They discussed the binary nature of assumptions which were a limiting factor in designing and proving compliance. Interviewees used terms such as *“the regulations forbid you” (NPOL1), “you can’t assume” (HBUIL1)*, and *“it doesn’t allow” (INS3)*. The assumptions they were referring to include occupancy profiles, window sizes, window opening types, time and temperature at which windows can be assumed open, the use of fans to create air movement, and assumptions around when shading can and cannot be used *(CON1, CON2, INS2, GUI2, NPOL1, HBUIL1)*.

Some participants raised the way in which the TM59 methodology was introduced and integrated with approved calculation tools (including IESVE,^6^ TAS^7^ and DesignBuilder^8^) was a barrier to effectiveness. This was because of the lack of skills and modelling systems supporting passive and active overheating solutions *(LPOL4, INS2, CON1, MANF1, MANF2)*. One participant explained, *“the modelling process is possibly in its infancy. There is no formal accreditation process for a TM59 modeller and therefore it is difficult to identify competent modellers within the industry unlike with SAP and EPC energy modelling, which does have an accreditation process” (INS2)*.

A few participants outside of the construction industry made the insight that TM59 was not the right calculation methodology to test compliance because it was originally created to inform design, but has subsequently been adopted as a policy compliance requirement *(NPOL1, GUI1, LPOL4)*. A policymaker explained *“it [TM59] was never created for compliance and therefore might not be the best tool to demonstrate overheating compliance” (NPOL1)*. The same participant explained how compliance tools have been confused in the past. In reference to Part L and SAP, they explained, *“people use SAP and the EPC process as a proxy for design. SAP is used as a design tool and it was never meant to be that” (NPOL1)*. This illustrates the complexity of how design and compliance modelling has been confused in the past.

In applying the TM59 methodology and Part O requirements, many participants within the construction industry referred to specific assumptions which were not realistic of how residents used their homes. Some felt that the occupancy profiles in TM59 that must be used to demonstrate compliance resulted in the delivery of homes which were at higher risk of overheating. Interviewees raised the concern of high occupancy homes, such as families, as an issue which can increase overheating risk *(CON2, CON3, GUI2)*. As one interviewee explained “*the theory of assessing buildings and what happens in reality and who’s inside these buildings is not realistic. You’re currently restricted on how many people to assume inside a flat, when maybe our most vulnerable people might be … a larger family occupying a smaller space. It obviously wouldn’t pass” (CON3)*. The participant was suggesting that the number of occupants was underestimated; the true number of occupants would create more heat within rooms and lead to higher overheating risk, but that this is over-looked in the modelling assumptions.

Interviewees also identified that assumptions within Part O for passive cooling solutions were not representative of how they might be used in reality *(NPOL1, CON2, CON1, LPOL4, CON3, MANF1, HBUIL1, HBUIL3)*. Part O sets requirements for when windows should be assumed to be open or closed, which are more prescriptive than in the TM59 modelling methodology. The requirements specify windows must only be fully open when internal temperatures exceed 26 °C, they are assumed to be partially open between 22 and 26 °C, and fully closed when the internal temperature is below 22 °C (Part O, 2021, p. 8). Participants, both designers and developers, identified that this was not realistic of how occupants would operate homes. *“It’s not really how people live, people don’t operate their homes like a robot”* an architect said, referring to window opening behaviours which align with specific temperature set points *(CON2)*.

Manufacturers and installers of passive and active technologies which address overheating also felt that some technologies were not adequately represented within modelling assumptions and tools. Within Part O, internal blinds cannot be used as shading devices in the modelling assumptions, however external blinds and shutters can be used (Part O, 2021). A shutters and blinds manufacturer explained: *“modelling software, such as IESVE, assume zero ventilation for external shading calculations … this does not happen in practice, there are lots of blinds with different openness factors that allow for air flow while still blocking solar gains” (MANF1)*. Another manufacturer (who sells ventilation and cooling) explained that SAP modelling for Part L does not allow for new innovative technologies. They were referring to heat pump technologies which can recover heat from solar thermal and/or hot water sources to deliver a small amount of additional cooling (*“peak lopping”*) with a mechanical ventilation system *(MANF2)*. They explained *“what we sell, exists in a grey area, you can’t put the energy use into SAP, because SAP isn’t built for it. Even after speaking with Elmhurst*^9^ *you must enter it as cooling, and once you enter it like that any development will instantly fail” (MANF2)*. The impact of compliance modelling tools not supporting passive shading, or efficient innovative technologies, can lead designers to less sustainable or more energy intensive solutions *(MANF1, MANF2)*.

Some participants also raised the point that Part O restricts the use of neighbourhood scale interventions. This was perceived as a barrier to delivering passive shading *(NPOL1, GUI2, GUI1, HBUIL2)*. Green infrastructure was raised by multiple interviewees as a favoured solution to deliver shading and provide relief from the urban heat island effect. It was also preferred since it can deliver co-benefits for the community including flood resilience and improved health and wellbeing *(GUI1, GUI3, INS1, CON2, NPOL1, LPOL1, LPOL4)*. However, a representative within national policy explained *“Part O of the building regulations currently forbids you from counting any shading from trees” (NPOL1)*. To address this, one participant identified that more evidence was required to inform modelling for green infrastructure, *“we need to create guidelines and clarifications for urban morphologies, looking at how microclimate and urban design can influence wind routes and where green is more effective. But it’s very difficult because there isn’t a clear scientific way to deal with that yet” (GUI2)*.

The same issue was raised in the use and application of ceiling fans *(HBUIL1, RES1, CON1, LPOL1)*. A housebuilder explained, *“Part O doesn’t allow for the modelling of a ceiling fan, the effects of that can be quite substantial without implementing active cooling,”* referring to the comfort fans can provide to an occupant *(HBUIL1)*. A designer explained that the *“dynamic modelling software currently takes quite a conservative approach with fans in terms of the benefits they make, they only look at resultant operative temperature. However, the benefit of fans is that it blows air at you, evaporates [sweat] and cools you down. So, the benefit could be more” (CON1)*. Experts working on further developments of TM59 agreed that more evidence and modelling could better inform the next iteration of the methodology to better support the use of neighbourhood shading and air movement to improve comfort *(CON1, GUI2)*.

For these reasons, many participants felt the limitations in assumptions and modelling within Part O and TM59 currently pushed them towards cooling, despite the regulations supporting passive design technologies over active cooling (V). These included the restrictive assumptions for window openings, fans, internal blinds, external shading and neighbourhood green infrastructure, as well as the way they are represented within modelling tools *(CON1, CON2, CON3, NPOL1, MANF1, HBUIL1, HBUIL3)*. Participants who delivered housing projects felt the only way to meet the Part O requirements, with the restrictions in passive design assumptions, was to deliver new build homes with active cooling, particularly in urban areas *(HBUIL1, CON1)*. One developer explained, *“Part O is counter-productive and is currently pushing you down the route of cooling” (HBUIL1)*. Some participants expressed confusion over the conflicting messages within Part O, which supports passive design and states “mechanical cooling may only be used where insufficient heat is capable of being removed from the indoor environment without it” (Part O, 2021). As one participant explained, *“there needs to be a more definitive idea [from government] on how homes should be delivered. At the moment, it isn’t clear, and the current route taken isn’t really working” (INS1)*. Another participant explained how Part O was released alongside an update to the Part L regulations which introduces a minimum design efficiency for active cooling (Part O, 2021). This is the first time a minimum efficiency for active cooling has been introduced for residential buildings, which a few participants saw as inconsistent with a strategy to avoid active cooling use which is set out within the Part O requirements *(INS1, INS3)*.

### 4.2 Unrealistic for future proofing

Aside from the specifics of the technologies, there was a concern over whether the regulations were realistically futureproofing new builds and existing homes for future extreme summers.

Many participants believed that homes should be designed for future generations, which was currently not a requirement for the Part O regulations *(CON2, INS3, GUI1, HBUIL1, LPOL4, NPOL1)*. One interviewee explained, *“currently … we don’t mandate modelling for future climate scenarios* – *it’s just for 2020 weather files. So we’re building, but not for the future” (GUI1)*.

With regards to delivering homes for rising temperatures in the future, there were a mixture of views towards active cooling. Many participants felt it was not the most sustainable solution, and passive solutions should be encouraged and used *(CON1, CON2, CON3, HBUIL1, HBUIL2, HBUIL3, GUIL2)*. However, one participant noted that *“we’ll get to a point where it will get too hot, and passive measures will only do so much”*, implying that active cooling may become a normal part of UK homes *(LPOL4)*. Some participants recognised that active cooling may be required, particularly for urban areas where design restrictions including noise and security limit the ability to open windows and allow in nighttime ventilation to passively cool homes *(LPOL4, MANF2, MANF3)*.

Participants also raised that much of the future housing stock are existing homes which were not designed for overheating resilience, and are not covered by Part O *(GUI1, GUI2, RES1, HBUIL1, HBUIL2, CON1, CON2)*. One participant explained *“80% of the 2050 housing stock is already built … the new regulations are great, but we don’t have regulations for non-residential, or for existing buildings” (GUI1)*. Another participant looked to government as a call for action, *“enough has been done on new buildings, there are regulations, and we can adapt our designs, but government must look to existing buildings to limit overheating” (HBUIL1)*.

### 4.3 Flexibility and streamlining to overcome conflict and deliver high quality homes

In addition to specific issues within Part O, interviewees raised both the issue of conflict between different parts of the regulations as well as how they are enforced as challenges to delivering high quality, overheating-resilient homes.

Participants identified that design requirements set out in different parts of the regulations do not conform with one another. As one participant explained, *“Part O doesn’t really conform with Part L, or Part F in certain circumstances” (INS1)*. The restrictions to window openings within Part O were raised as an example of conflicting requirements which clash with Part F (ventilation) and Part K (safety) of the building regulations *(CON2, HBUIL1, INS1)*.

This conflict in design requirements is not only experienced at national policy, but also local policy. Delivery of new build homes and other buildings must consider local planning requirements in their proposed designs. These requirements often set out additional requirements for local pollution, acoustics, daylight, and views out, to achieve local planning approval. One housebuilder described how all these requirements trade-off against one another, *“it’s a seesaw right now* – *daylighting, overheating, energy and the three don’t balance. And then sometimes another curveball is noise” (HBUIL1)*. All these considerations impact on glazing area and window opening size which makes it difficult to deliver best practice across all requirements. However, often the way in which these regulations are enforced meant that housebuilders found it difficult to meet all the requirements and still deliver a good quality home *(HBUIL1, CON2)*.

Fire Safety, Approved Document B, was also raised as a barrier to external shading in building design. Some felt that the regulatory and cultural changes after the Grenfell disaster in London, led planners and building control to become *“overly cautious”* around fire safety and prevented them from delivering overheating resilience, another important part of long-term building safety *(MANF1, HBUIL2, HBUIL3)*. Grenfell was partly caused by the attachment of materials to the outside of a building that were unsuitable, but technically compliant with the regulations. In response, regulations for external materials must pass a more stringent testing and approval process. However, participants experienced this as a barrier to external shading. One housebuilder explained, *“finding a supplier that had non-combustible elements from a European supply chain in a country where it is tightly regulated was quite an issue, we did a lot of testing” (HBUIL3)*. While many housebuilders engaged within this study preferred the use of passive measures including external shading and were prepared to go the extra mile *(HBUIL1, HBUIL2, HBUIL3, HBUIL5)*, an interviewee explained these additional barriers may deter some developers due to the risk involved in not achieving planning approval and the additional cost and time required *(HBUIL3)*.

To improve this, interviewees asked for some more flexibility for how regulations are applied to manage these trade-offs and support innovation in overheating design *(HBUIL1, HBUIL2, HBUIL3, CON2, CON3)*. A housing association explained *“there needs to be more flex in how regulations and planning is applied. To deliver true sustainability you need to be able to balance design requirements and consider new ways of doing things” (HBUIL2)*. However, to deliver this, a representative from local policy identified *“the skills are currently substandard”* in reference to both industry and policy *(LPOL4)*. To ensure the flexibility is well meaning and to deliver high quality homes, participants felt that ups-killing was required across the industry to include planners *(HBUIL3, INS1)*, designers *(NPOL1, CON2)* and building control *(LPOL4, CON4)*.

More broadly, it emerged that some participants considered the building regulations as *“bare minimum”* in prescriptive design standards for future ready homes *(CON2, NPOL1)*. A few interviewees felt that housebuilders should be going beyond this minimum standard *(CON1, CON2, NPOL1)*. However, both those whose job it is to influence housebuilders, and housebuilders themselves, agreed there was an overwhelming amount of complexity of the building regulations. As such, the need to comply with multiple complex requirements associated with national policy, local policy and insurance clauses, has resulted in a culture of meeting the minimum requirements as the standard *(HBUIL2, HBUIL3, INS1, CON2)*. One interviewee explained, *“developers are thinking of lots of different things when planning land” (INS1)*. From a designers’ point of view, they explained *“once you convince your clients to do X for environmental reasons, Y for health and safety, Z for equity and affordability, it is very hard to convince your clients to push for best practice on everything due to the cost associated with it” (CON2)*. However, all housebuilders agreed that if modelling and design assumption barriers could be overcome, they would ideally use passive measures to go beyond the minimum Part O requirements *(HBUIL1, HBUIL2, HBUIL3, HBUIL4, HBUIL5)*. This was considered more cost effective since they *“stay around for a long period of time and will always deliver long-term value” (HBUIL2)*.

A few participants also raised that even if regulations are complied with during the design phases of a project, it may not deliver high quality homes *(LPOL4, CON4, INS1, INS3)*. This is because the design proposals that are presented for compliance is not how the building is constructed and delivered in practice. This includes the quality of the construction which may have defects associated with its passive and active systems. This issue was raised regarding overheating specifically, as well as broader design goals to deliver zero carbon, decent, comfortable and affordable homes *(CON4, LPOL4)*. Building control was described as a *“tick box”* exercise for government accreditation systems including Trustmark and Microgeneration Certification Scheme (MCS) not leading to high quality outputs *(LPOL4, CON4, INS2)*. One participant who delivers retrofit and new build schemes explained, “*the current approach is about paperwork and things done behind a computer rather than the deeply rooted fundamentals of doing the job right.”* To overcome this, they went on to say, *“government need to employ building inspectors to come to site and audit in person rather than an obsession with paperwork, we need that practical approach” (CON4)*.

## 5 Discussion

This paper explores the application of Part O of the English buildings regulations in designing comfortable and sustainable homes for rising summertime temperatures. The study documents the lessons learnt from construction and policy professionals in the built environment. While Part O was well received and considered a positive action for overheating and building design, many interviewees shared barriers in its application towards achieving sustainable solutions to overheating. This paper presents empirical evidence from interviews with experts in the construction industry and government. In response it makes recommendations for future revisions of Part O of the regulations.

Many of the barriers identified by participants evidenced the binary approach for design requirements and compliance modelling assumptions set within the approved document Part O. This includes modelling assumptions for passive and active technologies, occupancy assumptions, modelling software assumptions, and the way in which the modelling methodology is used and applied. In the near-term, the next iteration of Part O, which is currently being revised, could address some of these conflicts.

There is potential to review the TM59 criteria for overheating. A more flexible criteria could support more use of passive solutions and flexibility in how designs are delivered. For example, Sadeghi et al. (2024) suggest alternative metrics, which may be better suited to variations in comfort with the inclusion of occupancy clothing, exposure to heat and length of heat events. In addition, Lomas and Li (2023) suggest replacing the current night-time temperature threshold of 26 °C with a more flexible threshold between 26 °C and 29 °C based on housing archetypes and resident’s needs. This is similar to the French regulations, which introduced a flexible internal comfort threshold between 26 °C and 28 °C (RE-2020, 2024).

Some participants perceived barriers in the assumptions for passive technologies and the lack of flexibility in how occupants are represented. This is something which Chen (2019) also raised as a risk and identified the need for greater flexibility in occupancy assumptions and passive technology to manage the reliance on active cooling. To address this, modelling assumptions could be revised in TM59. This could include assumptions for night ventilations, fans, shading and occupancy profiles, issues which interviewees have raised in this study.

A few interviewees felt that with temperatures increasing, active cooling will be necessary to deliver comfort and mitigate health risks. Recent survey data shows this may already be the case, with the uptake of active cooling increasing much faster than originally thought (Khosravi et al., 2025). To manage energy and resources, future revisions for Part L should explore the potential for regulating active cooling more tightly, particularly around the refrigerants used and product standards and efficiencies (Hoggett et al., 2024). However, the need to integrate this with broader net zero transitions to maximise passive design and reduce cooling loads, is critical and recognised by others who have researched this topic (Elsharkawy and Zahiri, 2020; Howarth, 2024; Hoggett, 2025).

Interviewees also identified that the regulations should ensure all homes (both new build and existing) are suitable for future warming climates. If homes continue to be designed for 2020 weather files, interviewees felt they would be inadequate to rising temperatures and may lead to increased active cooling loads. To address this, this paper suggests future Part O revisions should require modelled performance against the 2030, 2050 and 2080 weather files (CIBSE, 2025a,b). This would not be the first part of the building regulations to consider future climate within the design. For example, Part H (Drainage and waste disposal) sets a requirement to ensure all below ground rainwater drainage systems are designed for a 1-in-100 year flooding event + 40% climate change allowance to account for future flood risk (CIRIA, 2007; Part H, 2015). In addition, Part O regulations should extend to existing buildings, something the Climate Change Committee also recommended to manage heat risk in the existing housing stock (CCC, 2022). There is already precedent for this; Part L was initially introduced in 1995 for new buildings and later revised in 2005 to include existing dwellings where works are carried out over 25 m^2^ (Part L, 1995; Part L, 2005).

Beyond regulatory shifts, policy instruments could support retrofit of existing buildings for both net zero and heat resilience. Options could include extending existing grants for retrofit, which is already emerging. For example, the Warm Homes Plan extends funding to passive cooling measures including external shading and shutters (DESNZ, 2026). In addition, the Boiler Upgrade Scheme provides grants for air-to-air heat pumps with cooling (BUS, 2022). The Climate Change Committee suggest policy levers for social housing could include grants linked to Decent Homes Standard, and for owner occupied homes incentives associated with green mortgages or stamp duty (CCC, 2022). Funding grants or training opportunities to support skills across the retrofit supply chain should also be provided to integrate heat risk with retrofit for the decarbonisation of heat (Hoggett et al., 2024).

Beyond the specific design assumptions driven by Part O, interviewees raised conflicts with broader parts of the regulations as a challenge. This includes Part F (Ventilation), Part L (Conservation of Fuel and Power), Part B (Fire Safety) and Part K (Falling, Collisions and Impact), Part E (Noise). In the longer-term, opportunities to integrate overheating design with design to prevent heat loss and decarbonise heat is needed; this is something others have identified (EAC, 2024; Howarth, 2024). In building codes around the world where there is a heating and summer season, overheating is a part of the low carbon building design (CALGreen, 2023; RE-2020, 2024). This paper therefore recommends a future streamlining of Part O and Part L with the use of HEM to do this. This modelling methodology has already been created and is soon to be released for Part L compliance.

Integrated use of HEM would overcome challenges which interviewees identified in modelling. HEM was developed as an opensource tool to address some of the same concerns interviewees raised within this study. These include the need to be more flexible in design assumptions and incorporate new technologies easily (HEM, 2023c). While it is currently not proposed for overheating compliance, it can carry out detailed overheating calculations (HEM, 2023a). HEM also allows for future expansion, in a modular way, to include overheating analysis using the TM59 methodology in the future. The calculation methodology includes the ability to account for shading from neighbouring buildings, allowing for the addition of possible shading from green infrastructure, another barrier identified by interviewees in this study. In addition, the HEM methodology highlights the need to adjust occupancy profiles for hot water energy use (HEM, 2023a). This shows how HEM could support a shift in how occupancy profiles are applied for Part O, which could represent a more diverse set of households. The ability to use the same tool for both Part L and Part O of the regulations could reduce conflict between regulations and address multiple issues together in the future. This has added benefits of streamlining building regulations calculations and minimising duplication of processes for developers and designers.

Another finding which emerged from the interviews was that, even if the approved documents provide flexibility in how the regulations are met, interviewees across all sectors including designers, developers and even policymakers generally interpreted the approved document as minimum standards and assumptions that “must” be used, or are “required” to be met. This suggests that flexibility which already exists in the regulations may not be applied in practice. More broadly, interviewees raised that even if buildings are designed to the regulations and meet “minimum standards” or overcome design conflicts, it may not translate into the delivery of high-quality homes due to the culture and current approach to assessing building control.

To support delivery of high-quality homes, a new approach focusing on outputs, rather than prescriptive design requirements, may address these barriers. This could also encourage innovation, streamline current processes and alleviate conflicts between regulations which is needed to deliver future-ready buildings. This paper therefore proposes a new approach to Part O which focuses on the construction quality, and occupational period of the home, delivering long-term comfortable, affordable and sustainable homes. This may include setting standardised targets for internal comfort, such as an absolute internal temperature limit and annual energy use associated with active cooling. The proposed approach would create a process to ensure buildings focus on construction quality and post occupancy evaluation through audits during building delivery and occupation. Long-term post occupancy evaluation can better close the performance gap and ensure that buildings are delivered as they are designed, and that any defects are addressed during occupation (RIBA, 2020; Ford et al., 2022). This is suggested as a long-term shift in the regulations.

Although this is a radical shift in building regulations, this may be the best way to create a cultural shift in how buildings are designed and constructed, which is required to deliver heat resilient and net zero homes. Creating a new system would alleviate risks and carry-over in how the current system is applied. A focus on construction audits and post-occupancy evaluation could reshape the responsibilities of the sector, as design would be tied to long-term performance objectives, creating a strong incentive to close the performance gap. Performance-based compliance is emerging for non-domestic buildings through independent quality assurance standards. This includes LETI^10^ and UKNZCBS.^11^ Interviews were all carried out by the primary researcher which were recorded, anonymised and transcribed to minimise operational carbon and whole life carbon (LETI, 2020; RIBA, 2020; UKNZCBS, 2025). They set absolute limits which must be met during design, and subsequently monitored and compared after construction to achieve compliance. The Greater London Authority also ask for in-use data to ensure buildings are performing as intended (Greater London Authority, 2021).

To apply to overheating, this approach would require further evidence, particularly to identify robust metrics for a variety of homes, climates and local needs. This is something the Department of Energy Security and Net Zero (DESNZ) and the Health and Safety Executive (HSE) are already working on to explore options for climate resilience metrics (CCC, 2025, p. 75). UKNZCBS stress the need for robust modelling approaches completed by experienced professionals to ensure quality and robustness (UKNZCBS, 2025). Use of HEM as a compliance tool could be an opportunity to revise and address the modelling methodology for overheating to deliver this robustness.

In delivering a policy shift such as this, it must also be in tune with current systems and processes used within industry to ensure the system still upholds quality and does not introduce additional inefficiencies in cost and time. Further evidence should be gathered to understand these conflicts between Part O and other parts of the regulations. Case studies which monitor live building projects could evidence conflicts design teams are facing throughout the design process, during construction and occupation. These can then be addressed either within revisions of the building regulations (in the near or long term), or by supporting skills development for those who use and enforce the regulations to help inform these trade-offs and manage negotiations. In addition, evidence of specific technologies, including the performance of green infrastructure, fans and shading to deliver cooling effects will continue to inform more detailed assumptions both for design and compliance purposes.

## 6 Conclusion

This paper explores the application of Part O of the English buildings regulations in delivering heat resilience for homes across England. This study gathers empirical evidence from the experiences of those in the construction and policy sector to understand barriers in delivering overheating-resilient homes. In exploring these barriers, this paper provides recommendations to improve the application of Part O and wider building regulations to deliver high quality, comfortable and affordable homes.

This paper recommends near-term improvements to Part O in future revisions with informed updates to passive and active modelling assumptions, in addition to the TM59 modelling methodology. This should also require all homes, both new and existing, to be designed to future weather to ensure they are resilient to rising temperatures. In the long-term, enabling a shift towards passive technologies which align with the net zero transition and goals to deliver high quality homes may require broader shifts in the building regulations. This could include the use of HEM to streamline modelling approaches and in addition address many of the overheating modelling challenges identified in this study. It could also include a shift in how overheating is regulated, to move towards performance-based metrics, with audits and post-occupancy evaluation to ensure compliance. To support shifts in the building regulations, policy must also support the upskilling of both policy and construction professionals. In addition, retrofit for heat resilience should be incentivised through grants or pricing mechanisms for passive and active measures which can deliver heat resilience alongside net zero.

## Figures and Tables

**Table 1:** Summary of interviewees engaged with, and their relevant sectors.

Interview Group	Term	Number of Participants
Industry associations	GUI	3
Building design consultancy	CON	4
Social researchers	RES	1
Housebuilders and housing associations	HBUIL	5
National policy	NPOL	3
Local policy	LPOL	5
Building insurance	INS	3
Housing and health charities	CHAR	2
Manufacturers and installers	MANF	5
		30

## Data Availability

The authors do not have permission to share data.

## Footnotes

1 TM59 – Technical Memorandum 59: Design methodology for the assessment of overheating risk in homes (2017).

2 TM52 – Technical Memorandum 52: The limits of thermal comfort: avoiding overheating (2013).

3 UKCP09 – UK Climate Projections 2009

4 UKCP18 - UK Climate Projections 2018

5 European Union.

6 IESVE – Integrated Environmental Solutions, Virtual Environment.

7 TAS – Thermal Analysis System.

8 DesignBuilder Software Ltd (which uses EnergyPlus).

9 Elmhurst are an accredited software provider which is used to complete SAP calculations and create EPCs for new build and existing homes.

10 LETI – Low Energy Transformation Initiative.

11 UKNZCBS – United Kingdom Net Zero Carbon Buildings Standards.
